# Can proline dehydrogenase—a key enzyme involved in proline metabolism—be a novel target for cancer therapy?

**DOI:** 10.3389/fonc.2023.1254439

**Published:** 2023-11-06

**Authors:** Xiangyuan Xu, Guangtao Zhang, Yijia Chen, Weina Xu, Yujing Liu, Guang Ji, Hanchen Xu

**Affiliations:** ^1^ Institute of Digestive Diseases, Longhua Hospital, Shanghai University of Traditional Chinese Medicine, Shanghai, China; ^2^ Shanghai Frontiers Science Center of Disease and Syndrome Biology of Inflammatory Cancer Transformation, Shanghai, China; ^3^ Department of Gynecology, LongHua Hospital, Shanghai University of Traditional Chinese Medicine, Shanghai, China; ^4^ Shanghai Pudong New Area Zhoujiadu Community Health Service Center, Shanghai, China

**Keywords:** ProDH, proline metabolism, cancer therapy, cancer metabolism, immunosuppression

## Abstract

Emerging evidence suggests that proline metabolism is important for regulating the survival and death of different types of cancer cells. Proline dehydrogenase (PRODH), an enzyme catalyzing proline catabolism, and the degradation products of proline by PRODH, such as ATP and ROS, are known to play critical roles in cancer progression. Notably, the role of PRODH in cancer is still complicated and unclear, and primarily depends on the cancer type and tumor microenvironment. For instance, PRODH induces apoptosis and senescence through ROS signaling in different types of cancers, while as a protumor factor, PRODH promotes malignant phenotypes of certain tumors under stresses such as hypoxia. In order to assess whether PRODH can serve as a novel target for cancer therapy, we will provide an overview of the biological functions of PRODH and its double-edged role in cancer in this article.

## Introduction

During tumor progression, malignant cells make certain adjustments to energy metabolism to gain enough energy for supporting their uncontrolled proliferation. Therefore, the 2011 edition of the Hallmarks of Cancer introduced the concept of reprogramming energy metabolism (1). In fact, as early as the 1920s, Otto Warburg has already observed an abnormality in the metabolism of cancer cells, whereby they heavily rely on glycolysis as their primary energy metabolism pattern even when they are well oxygenated, thus showing a high rate of glucose uptake and lactate secretion, which is known as aerobic glycolysis or the Warburg effect (2).

In addition to glucose, which is heavily consumed during aerobic glycolysis, high quantities of glutamine are also internalized by tumors. Therefore, deregulated uptake of glucose and glutamine is one of the important characteristics of cancer metabolic reprogramming (3, 4). Apart from glutamine, cancer cells also consume other amino acids such as serine, glycine and proline to generate nucleotides, reactive oxygen species (ROS), proteins and oncometabolites (5).

It is widely recognized that amino acids can be categorized into essential amino acids (EAAs) and nonessential amino acids (NEAAs) based on dietary necessity. The important and diverse roles of NEAAs in tumor metabolism have spurred the development of related therapies (6). For example, the depletion of blood asparagine by using asparaginase is involved in modern clinical treatments of childhood acute lymphoblastic leukemia (ALL) because ALL cells are in great need of exogenous asparagine (7).

As one of the NEAAs, proline also plays a critical role in cancer due to metabolic changes (8), according to the previous discoveries, it is evident that proline and its metabolic enzymes plays a multifaceted role in cancer. In recent years, a series of new findings have also reported on this. Thus, we aim to summarize these relevant studies and elucidate how proline metabolism and the key enzymes involved in proline metabolism influence cancer progression.

## Proline and proline metabolism

Proline originates from various sources, including dietary intake, endogenous synthesis from other amino acids within cells, and the degradation of proline-rich proteins like collagen. The imidodipeptides generated from protein degradation are cleaved by prolidase and prolinase, resulting in the liberation of proline and hydroxyproline (9).

For mammals, one of the functions of proline is to protect cells from damage caused by oxidative stress and preserving redox homeostasis, consequently, mammalian cells will enhance proline biosynthesis in response of oxidative stress (10, 11). Besides, proline plays a critical role in stabilizing the structure of collagen as one of the main constituents. Collagen serves as the most abundant component of the extracellular matrix(ECM), provides support for cell growth (12).

**Figure 1:**
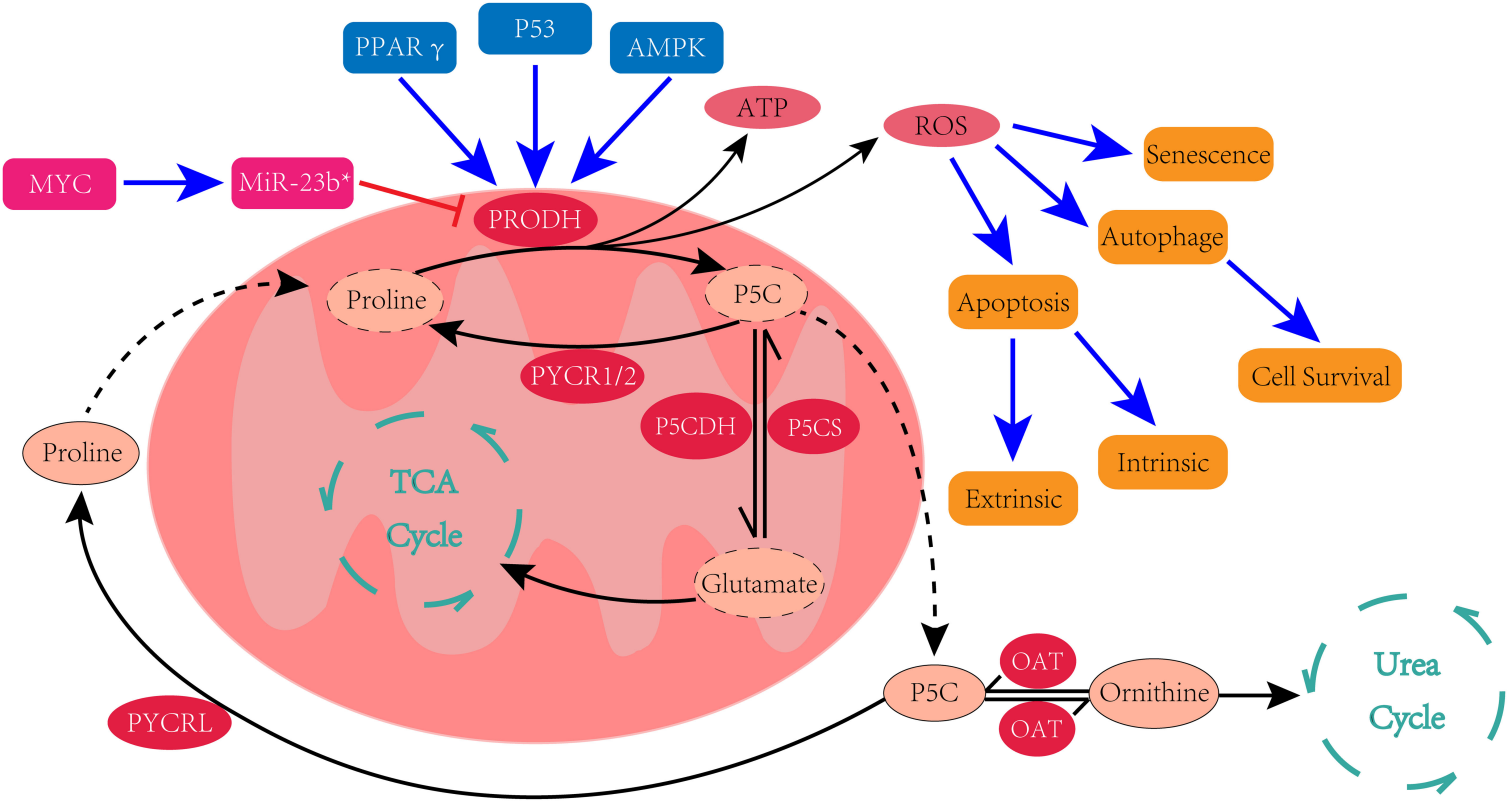
Proline metabolic pathway and the regulators of PRODH.

The unique structure of proline renders its metabolic pathways distinct from most amino acids, as the secondary amino group of proline precludes its catalysis by transaminase. Therefore, the metabolism of proline necessitates the existence of an intermediate product, rather than proline itself, as the center of its metabolic pathways. Early studies have proved that proline can convert to glutamate and ornithine, and can also be synthesized from glutamate and ornithine. Δ^1^-pyrroline-5-carboxylate (P5C) serves as the central intermediate in the metabolic interconversions between these three amino acids (13, 14) (**Figure 1**).

### Proline biosynthesis

As mentioned above, glutamate and ornithine can both serve as substrates for proline biosynthesis. Both glutamate and ornithine are first converted to glutamate-γ-semialdehyde (GSAL) rather than directly converted to proline. GSAL serves not only as the precursor of proline but also in tautomeric equilibrium with P5C (14), meaning that GSAL and P5C are tautomers in dynamic balance that can spontaneously interconvert. Despite this understanding, the underlying factors that determine whether P5C or GSAL is the intermediate product remain to be elucidated (15).

Glutamate is converted into GSAL/P5C by P5CS (Δ^1^- pyrroline-5-carboxylate synthase), a mitochondrial enzyme encoded by the ALDH18A1 gene. The function of P5CS is to phosphorylate glutamate and reduce it to P5C subsequently in an ATP and NAD(P) H-dependent manner (16). In contrast, the generation of GASL from ornithine is catalyzed by ornithine aminotransferase (OAT).

The final step of proline biosynthesis is the conversion of P5C to proline, which is catalyzed by Δ^1^-pyrroline-5-carboxylate reductase (PYCR). There are 3 separate genes responsible for encoding homologous PYCR enzymes: PYCR1, PYCR2 and PYCR3/PYCRL. PYCR1 and PYCR2 use P5C formed by P5CS as the substrate to generate proline and need NADH as the cofactor, whereas PYCRL generates proline via P5C derived from ornithine and prefers NADPH as the cofactor (17). Despite the fact that these three PYCR isoforms have differences in structure, location and function, they play an unequivocal role in promoting the development of several cancers (18). PYCR1 is the most studied among them. Moreover, emerging evidence suggests that PYCR1 is upregulated and plays a protumor role in several different types of cancers, including non-small cell lung cancer (19, 20), breast cancer (21), gastric cancer (22), colorectal cancer (23), hepatocellular carcinoma (24, 25), prostate cancer (26) and bladder cancer (27).

### Proline catabolism

The first step of proline catabolism is catalyzed by PRODH. The PRODH enzyme is located on the inner mitochondrial membrane, where it catalyzes the oxidation of proline and converts it into P5C in a FAD-dependent manner. FAD is then reduced to FADH2 during this enzymatic reaction, and the electrons from the reduced FAD are transferred to the mitochondrial electron transport chain (ETC) for ATP production or participate in the generation of ROS (28).

Indeed, there are two PRODH enzymes, the more accurate description of the enzyme mentioned above is PRODH1, and the PRODH1 gene, which encodes the enzyme, is located on chromosome 22q11.2. PRODH2, which catalyzes the oxidation of trans-4-hydroxy-L-proline to Δ^1^-pyrroline-3-hydroxy-5-carboxylate (3-OH-P5C), is encoded by the PRODH2 gene located on chromosome 19q13.12 (29). Although they exhibit a high degree of similarity in both amino acid sequence and active site sequence (30), the biological differences between them are evident. In fact, few studies have focused on PRODH2 compared with PRODH1 at present, but a recent study identified that knock-in and overexpression of PRODH2 can significantly improve the antitumor effects of chimeric antigen receptor T-cell (CAR-T) immunotherapy (31), so perhaps PRODH2 is a potential target for enhancing CAR-T therapeutic efficacy. Nevertheless, PRODH in a broad sense refers to PRODH1, so PRODH will be used to refer to PRODH1 in this article.

The next step of proline catabolism can be simply regarded as the reversal of proline biosynthesis. Proline-derived P5C/GASL can be converted back to glutamate through the catalysis of Δ^1^-pyrroline-5-carboxylate dehydrogenase (P5CDH), a NAD^+^-dependent enzyme located in the mitochondrial matrix encoded by the ALDH4A1 gene (32), after which the produced glutamate is converted to α-ketoglutarate and enters the tricarboxylic acid cycle (TCA cycle). Alternatively, P5C/GASL can be converted to ornithine through the catalyzation of OAT and then participate in the urea cycle. The reversible conversions of P5C and ornithine establishes proline metabolism as the exclusive pathway linking the TCA cycle and the urea cycle (33).

PRODH and PYCR are the key enzymes involved in proline metabolism. Interestingly, compared to the unambiguous promoting effect of PYCR on tumorigenesis and cancer progression, PRODH plays a dual role in cancer. Some reports suggest that the function of PRODH as either a protumor factor or a suppressor of cancer can vary depending on the cancer types and the metabolic context (34). Hence, here in this article, we aim to elucidate the intricate mechanisms underlying the dual role of PRODH in cancer (**Table 1**).

**Table 1:** The double-edged role of PRODH in cancer.

Function	Mechanisms	Types of tumors
Anti-tumor	Induce apoptosis	Breast cancer (35) Colorectal cancer (36); Melanoma (37) Oral squamous cell carcinoma (38)
	Induce cellular senescence	Osteosarcoma (39)
Pro-tumor	Induce protective autophagy	Breast cancer (40) Colorectal cancer (41) (42)
	Promote metastasis and invasion	Breast cancer (43) Non-small cell lung cancer (44)
	Promote immunosuppression	Prostate cancer (45)
	Supply energy	Pancreatic ductal adenocarcinoma (46)

## PRODH as an antitumor factor

### Apoptosis

In 1997, researchers observed that PRODH is among the 14 genes that exhibit the most significant upregulation in response to p53-induced apoptosis, hence it’s also known as p53-induced gene 6 (PIG6) (47). This finding had great importance for subsequent studies investigating PRODH in human cancers. A follow-up study confirmed that PRODH is involved in the response to p53-mediated apoptosis (48). After further investigations into the mechanisms by which p53 regulates PRODH expression, it was discovered that the promoter region of PRODH contains a p53-responsive element, suggesting that p53 functions as a direct regulator of PRODH (49, 50).

In addition, multiple studies indicated that PRODH plays a proapoptotic role through generating intracellular ROS (51, 52). However, PRODH does not produce ROS directly; it drives ROS production by transferring electrons into the ETC and providing anaplerotic carbon for other mitochondrial dehydrogenases (53).

Due to the generation and accumulation of ROS, PRODH can induce both intrinsic apoptosis and extrinsic apoptosis (54). In the intrinsic (mitochondrial) apoptosis pathway, PRODH has been shown to trigger the release of cytochrome c into the cytosol from the mitochondria, leading to the activation of caspase-9 (55). This finding indicates the ability of PRODH to induce intrinsic apoptosis in a p53-independent manner. In addition to intrinsic apoptosis, PRODH has also been found to stimulate the expression of tumor necrosis factor-related apoptosis inducing ligand (TRAIL), thereby activating the extrinsic apoptosis pathway through the activation of caspase-8 (54). This upregulation of TRAIL is activated by nuclear factor of activated T cells (NFAT). Intriguingly, a previous study demonstrated that PRODH can activate the Ca^2+^/calcineurin pathway through the generation of ROS, leading to subsequent activation of NFAT and then similarly triggering the release of cytochrome c, ultimately resulting in apoptosis (56).

Subsequently, studies on PRODH-mediated apoptosis have focused on the apoptotic effect of PRODH on cancer cells and its underlying mechanisms. A study utilizing DLD-1 colorectal cancer cells revealed the role of PRODH in regulating cyclooxygenase 2 (COX-2) to induce apoptosis. Upregulated expression of PRODH resulted in the suppression of COX-2/prostaglandin E2 (PGE2) activity; however, this suppression was partially reversed by manganese superoxide dismutase (MnSOD) (36), a key antioxidant enzyme known to protect against oxidative stress and inhibit PRODH-induced apoptosis (52), indicating that the reduction in COX-2/PGE2 by PRODH was a result of increased levels of ROS. COX-2 is identified as a prognostic marker of poor patient outcomes in most cancers and has been implicated in promoting various malignant phenotypes of several cancer cells (57). So undoubtedly, COX-2 inhibitors, such as celecoxib, have manifested some antitumor effects, such as inducing apoptosis of cancer cells (58). Notably, a study conducted on the effect of celecoxib on oral squamous cell carcinoma suggests that celecoxib treatment triggers apoptosis of cancer cells by upregulating the expression of PRODH (38). Moreover, a recent study demonstrated that, in addition to celecoxib, other nonsteroidal anti-inflammatory drugs (NSAIDs) can also induce apoptosis in breast cancer cells. As a class of ligands and agonists of peroxisome proliferator-activated receptor-γ (PPARγ), NSAIDs can be used to upregulate PPARγ expression, leading to a subsequent increased expression of PRODH and PRODH-dependent apoptosis (59). Thiazolidinediones (TZDs) have emerged as the most extensively studied and widely used synthetic ligands of PPARγ, with troglitazone being a prominent member. By using MDA-MB-231 breast cancer cells deprived of estrogen receptor β(ERβ) or cultured in medium without estradiol to establish the triple-negative breast cancer (TNBC) model, troglitazone treatment has been demonstrated to induce PRODH-mediated apoptosis through activating PPARγ in TNBC. Moreover, the absence of estradiol or ERβ inhibits collagen biosynthesis, thus providing proline for PRODH to induce apoptosis by ROS generation (35). Clinically, TZDs are usually used as hypoglycemic agents to treat type 2 diabetes mellitus (T2DM) (60). Interestingly, metformin, the first-line medication used for treating T2DM, has also been found to induce apoptosis through PRODH. In C32 melanoma cells, metformin was found to stimulate the expression of PRODH by activating AMP-activated protein kinase (AMPK) and inhibiting collagen synthesis (37).

In summary, the proapoptotic effect of PRODH on cancer cells is primarily determined by the activation of upstream signaling pathways that induce high expression levels of PRODH and inhibition of collagen synthesis to provide adequate proline for PRODH-mediated ROS generation. All these findings highlight the crucial role of PRODH in the regulation of cell death.

### Senescence

Cellular senescence can serve as a defense mechanism against cancer, as it leads to permanent cell cycle arrest in response to endogenous or exogenous stressors, such as oncogene activation (61). Although stable cell cycle arrest can be induced by various cellular mechanisms, rendering it unsuitable as a sole marker, it remains a critical characteristic for identifying all types of cellular senescence (62). Over a decade ago, Liu and his colleagues discovered that PRODH significantly reduced tumor formation by inducing cell cycle arrest in the G2 phase, potentially through the mediation of the GADD (growth arrest and DNA damage inducible gene) family (63). However, this discovery does not prove a direct link between PRODH and senescence.

More recently, researchers have shifted their attention to the role of PRODH in cellular senescence. Using low concentrations of etoposide to cause DNA damage, senescence was induced in different cell lines, and researchers identified PRODH as one of four genes involved in senescence through differential transcriptomics analysis (64). The following study investigating the relation between PRODH and senescence revealed the role of PRODH in promoting senescence and DNA damage. However, this promotion effect can be attenuated by the ROS scavenger N-acetyl-L-cysteine (NAC), suggesting the involvement of ROS in PRODH-mediated senescence. Additionally, another study showed that L-tetrahydro-2-furoic acid (L-THFA), an inhibitor of PRODH enzymatic activity, effectively suppressed etoposide-induced senescence and ROS production, indicating that cellular senescence mediated by PRODH is dependent on its enzymatic activity (39).

## PRODH as a pro-tumor factor

### Autophagy

In the earliest stages of cancer, autophagy seems to play a restrictive role in limiting tumorigenesis. But in fact, accumulating evidence indicates that autophagy enables cancer cells to resist intracellular and extracellular stresses such as hypoxia, nutrient deprivation and oncotherapy in established tumors, thereby promoting cell survival and cancer progression (65).

In an early study investigating the cytotoxic effects of oxidized low-density lipoproteins (oxLDLs) on cancer cells, researchers found that PRODH expression is upregulated by oxLDL treatment through the activation of PPARγ and the upregulated PRODH expression triggers protective autophagy in several cancer cell lines exposed to the noxious effects of oxLDLs (41). Another study investigating the impact of proline metabolism on tumorigenesis in hypoxic environments showed that PRODH expression in cancer cells was induced by hypoxia through AMPK activation, which contributes to tumor cell survival by inducing protective autophagy that relies on PRODH-induced ROS. Furthermore, the combination of hypoxia and glucose deprivation leads to an additive increase in PRODH expression, proline will be catabolized by PRODH to provide ATP for cellular energy needs preferentially, which means that PRODH acts as a survival factor under such extreme conditions (42, 66). HDAC inhibitors, such as TSA/SAHA, have also been found to induce an increase in PRODH expression through AMPK activation in triple-negative breast cancer (TNBC) cells. During this process, TSA/SAHA can induce autophagy and apoptosis simultaneously, however interestingly, PRODH did not promote the antiproliferative effects of HDAC inhibitors on cancer cells, but induced protective autophagy and suppressed apoptosis instead (40).

### Metastasis and invasion

Metastasis constitutes the greatest number of deaths for over90% of patients with cancer (67). It has been reported that proline catabolism driven by PRODH promotes metastasis of breast cancer. Data from clinical specimens demonstrates a significantly higher expression of PRODH in metastases compared to primary breast cancer tumors. Additionally, the reduced level of proline in lung metastases isolated from the orthotopic 4T1 breast cancer mouse model provides further evidence supporting this finding. Strikingly, the formation of breast cancer-derived lung metastases *in vivo* was reduced by using L-THFA to inhibit PRODH activity, indicating that PRODH could be a potential target for inhibiting breast cancer metastasis formation (43).

Besides, epithelial–mesenchymal transition (EMT) is considered as one of the phenotypes used to assess the invasiveness and metastatic potential of tumors. During EMT, cancer cells gradually lose their epithelial characteristics and acquire mesenchymal features (68). Recent research has highlighted the importance of PRODH in the progression of non-small cell lung cancer (NSCLC). Compared to normal tissues, lung adenocarcinoma specimens exhibit elevated expression levels of PRODH at both mRNA and protein levels. Overexpressing PRODH in NSCLC cells promotes EMT by facilitating migration and invasion of cancer cells. Conversely, EMT and cell proliferation can be suppressed by inhibiting PRODH activity (44).

### Immunosuppression

Although there are few studies that investigate the influence of PRODH on immune function, those that have been conducted have revealed that PRODH does indeed have a regulatory role in immune responses to defend against pathogens in eukaryotes (69, 70).

However, a recent study indicated that PRODH can suppress T-cell infiltration in prostate cancer. A substantial quantity of P5C generated by high expression of PRODH in prostate cancer cells induces an increase in ROS generation and a decrease in cytokines and ATP production, which results in the suppression of T cell function. But of all these detrimental effects could be reversed by PRODH knockdown. Furthermore, this study conducted a xenograft model to validate their *in vitro* findings and found that upregulation of PRODH in animal models contributes to tumor growth and decreased T-cell infiltration, while PRODH knockdown accelerates CD3+, CD4+, and CD8+ T cells infiltration (45). Overall, this study provides novel insight into how PRODH impairs immune cell function.

### Supply energy

When confronted with nutrient stress, cells will break down cellular and tissue components to sustain their energy requirements. In such circumstances, proline catabolism assumes significance as cells can acquire proline by breaking down the extracellular matrix and subsequently initiate proline degradation through PRODH to yield ATThis degradative pathway also generates glutamate and α-ketoglutarate, products that can enter the TCA cycle. Furthermore, glucose deprivation increases the levels of intracellular proline and PRODH expression levels in cancer cells, leading to further activation of the pentose phosphate pathway. In light of these observations, it can be posited that proline and PRODH-mediated proline catabolism serves as one of the alternative mechanisms by which cells are able to maintain energy levels under nutrient stress (71).

In pancreatic ductal adenocarcinoma (PDAC), cancer cells exist in glandular structures surrounded by a collagen-rich meshwork that forms their ECM and lacks nutrients and oxygen. Under such nutrient-deprived conditions, PDAC cells have the capability to utilize proline derived from the degradation of collagen to maintain TCA cycle and ensure cell survival. Interestingly, even under nutrient-sufficient conditions, some degree of proline catabolism through PRODH appears to be necessary, as it promotes PDAC cell growth and survival *in vitro* and *in vivo*. Therefore, this study shows the possibility of slowing PDAC tumor growth and reducing tumor survival by specifically targeting proline metabolism through PRODH inhibition (46).

## Regulators of PRODH

### PPARγ

In studies screening potential regulators for PRODH other than p53, researchers found that PPARγ demonstrates the strongest capacity for activating the PRODH promoter among all the examined transcription factors, as other transcription factors only showed modest stimulatory effects. Subsequent experiments provided further confirmation that the PRODH promoter indeed contains a PPAR response element, and activated PPARγ is capable of binding to this region of the PRODH promoter (72).

After confirming the direct regulatory effect of PPARγ on PRODH, many studies have shifted their focus to how PPAR ligands exert their effects through PRODH in tumors. Some ligands of PPARγ occur naturally, such as prostaglandins and oxidized lipids, while others are synthetic, such as TZDs. Most studies have indicated that these ligands can not only activate the PRODH promoter but also upregulate PRODH expression and subsequently promote apoptosis in cancer cells by increasing ROS (59, 35, 73, 74). However, as mentioned above, PPAR-activated PRODH can also serve as a survival factor for cancer cells (41).

Interestingly, the role of PPARγ in tumorigenesis is also controversial. Although substantial evidence suggests that PPARγ activation has a positive effect on tumor suppression, PPARγ has also been implicated in promoting cancer progression in multiple types of tumors (75).

### AMPK

AMPK serves as a cellular energy status monitor by sensing the levels of AMP and ADP relative to ATP and becomes activated when a decrease in cellular energy levels is detected. In addition, AMPK is also capable of sensing the availability of glucose and can be activated by glucose deprivation (76).

A previous study investigated that AMPK could be activated by phosphorylation under glucose deprivation. After treating cancer cells with a synthetic AMPK activator, the activity of PRODH appears to have an increase (71). Additionally, another study found that the expression of PRODH under hypoxia is dependent on AMPK activation, rather than the critical transcription factors HIF-1α or HIF-2α, which are known to be overexpressed in response to hypoxia in cancer (42). However, it should be noted that the mechanisms underlying the regulation of PRODH by AMPK remain unclear and require further research and investigation.

### c-MYC

MYC is one of the earliest discovered oncogenic transcription factors, and the oncoproteins encoded by MYC are widely activated and expressed in human tumors. Moreover, MYC rewires multiple metabolic pathways to support tumor growth and cancer cell proliferation (77). In a study investigating the impact of c-MYC on PRODH, it was observed that high expression of c-MYC leads to a decrease in PRODH protein expression, but researchers found no evidence to support the direct binding of c-MYC to the PRODH promoter region. Therefore, they speculated that this process is not directly regulated by c-MYC but is instead mediated by c-MYC through regulating other transcription factors and then linked this hypothesis to their previous finding about the regulation of PRODH by microRNAs (miRNAs) (78).

MicroRNAs (mRNAs) can regulate target genes by inhibiting the translation and affecting the stability of target messenger RNAs (79). Therefore, microRNAs exert a certain influence on tumorigenesis and cancer progression. In an earlier study examining the regulation of PRODH by miRNAs, researchers found that in normal renal epithelial cells transfected with several different miRNAs, only miR-23b^*^ significantly inhibited PRODH protein expression, whereas in renal cancer cells, this effect could be reversed by an antagomir against miR-23b^*^. Together, these data indicate the suppression of PRODH by miR-23b^*^. Notably, in clinical specimens of renal cell carcinoma, the expression of miR-23b* is commonly upregulated (80).

Based on the aforementioned findings, researchers examined whether c-MYC can regulate PRODH through miR-23b^*^. As expected, c-MYC significantly upregulated the expression of miR-23b^*^, and the upregulated miR-23b^*^ suppressed PRODH expression (78). This outcome provides a partial elucidation of how c-MYC regulates PRODH.

## Conclusion and future prospects

Based on current research, it is evident that proline catabolism and PRODH are important parts of the tumor process. However, it should be noted that the specific role of PRODH may not be consistent across different types of tumors and pathological conditions. On one hand, the ability of PRODH to induce apoptosis and senescence in cancer cells reflects its role as a tumor suppressor. On the other hand, in some contexts, such as hypoxia, PRODH can also function as an oncogene by promoting cancer cell survival, growth, and metastasis.

Although closely related to the development of cancer, currently, there are no drugs available that specifically target proline catabolism or PRODH. Furthermore, another great challenge is that treatment strategies targeting PRODH require modifications depending on the type of cancer and metabolic context. Even in the same type of cancer, the role of PRODH is not singular or fixed. For instance, in breast cancer, high expression of PRODH promotes the apoptosis of cancer cells, but it also contributes to the formation of lung metastases. In this case, it is worth considering and discussing whether to inhibit or stimulate the activity of PRODH. Nonetheless, searching for pharmacological compounds that can target PRODH for cancer-targeted therapy is still of great importance. By observing the effects of these potential molecules on tumors *in vitro* and *in vivo*, we may gain a better understanding of the function of PRODH.

Another challenge in PRODH-related research is the dearth of breakthrough findings that would give us a clearer understanding of PRODH. Nonetheless, the abundance of literature in this field has increased in recent years, which has to some extent increased the amount of available information. Moving forward, the development and application of appropriate technical means to more thoroughly study the role of PRODH in cancer will be crucial.

In summary, PRODH is a potential therapeutic target and it’s necessary to conduct further in-depth research on it.

## Author contributions

XX: Writing – original draft. GZ: Writing – review & editing. YC: Writing – review & editing. WX: Writing – review & editing. YL: Writing – review & editing. GJ: Writing – review & editing. HX: Writing – review & editing.
